# The Involvement of *RhoA* and *Wnt-5a* in the Tumorigenesis and Progression of Ovarian Epithelial Carcinoma

**DOI:** 10.3390/ijms141224187

**Published:** 2013-12-12

**Authors:** Shuo Chen, Jun Wang, Wen-Feng Gou, Ying-Ling Xiu, Hua-Chuan Zheng, Zhi-Hong Zong, Yasuo Takano, Yang Zhao

**Affiliations:** 1 Department of Gynecology, the First Affiliated Hospital of China Medical University, Shenyang 110001, China; E-Mails: chenshuo077003@163.com (S.C.); xiu561480485@163.com (Y.-L.X.); 2 Department of Gynecology, the General Hospital of Shenyang Military Region, Shenyang 110045, China; E-Mail: wj202fck@163.com; 3 Department of Biochemistry and Molecular Biology, Institute of Pathology and Pathophysiology, College of Basic Medicine, China Medical University, Shenyang 110001, China; E-Mails: xiaogouaeiou@163.com (W.-F.G.); zheng_huachuan@hotmail.com (H.-C.Z.); zzh660526@sina.com (Z.-H.Z.); 4 Clinical Cancer Institute, Kanagawa Cancer Center, Yokohama 241-0815, Japan; E-Mail: ytakano@gancen.asahi.yokohama.jp

**Keywords:** ovarian carcinoma, *RhoA*, Wnt-5a signaling, tumorigenesis and progression, phenotype

## Abstract

**Background:**

Ras homolog gene family member A (*RhoA*) is involved in Wnt-5a–induced migration of gastric and breast cancer cells. We investigated the roles of *RhoA* and *Wnt-5a* in ovarian carcinoma.

**Methods:**

RhoA and Wnt-5a mRNA and protein expression in normal fallopian tube epithelium, benign tumors, primary ovarian carcinomas, and metastatic omentum were quantified. RhoA or Wnt-5a was knocked down in OVCAR3 ovarian carcinoma cells using siRNAs and cell phenotype and expression of relevant molecules were assayed.

**Results:**

RhoA and Wnt-5a mRNA and protein expression were found to be significantly higher in metastatic omentum than in ovarian carcinomas, benign tumors, and normal fallopian tube epithelium (*p* < 0.05), and positively associated with differentiation and FIGO staging (stage I/II *vs.* stage III/IV) in ovarian carcinoma (*p* < 0.05). *RhoA* and *Wnt-5a* expression were positively correlated in ovarian carcinoma (*p* = 0.001, *R*^2^ = 0.1669). RhoA or Wnt-5a knockdown downregulated *RhoA* and *Wnt-5a* expression; reduced cell proliferation; promoted G_1_ arrest and apoptosis; suppressed lamellipodia formation, cell migration, and invasion; and reduced PI3K, Akt, p70S6k, Bcl-xL, survivin, and VEGF mRNA or protein expression.

**Conclusions:**

This is the first demonstration that *RhoA* and *Wnt-5a* are associated with ovarian carcinogenesis and apoptosis inhibition; there might be positive correlation between *RhoA* and *Wnt-5a* expression. *RhoA* is a potential tumorigenesis, differentiation, and progression biomarker in ovarian carcinoma.

## 1. Introduction

Ovarian cancer is a malignant tumor that represents a serious threat to women’s health and is one of the leading causes of cancer-related deaths in females. More than 90% of ovarian cancers are classified as epithelial and are believed to arise from the ovarian epithelium [1]; however, evidence suggests that the fallopian tube inner surface epithelium may also be the origin of some ovarian cancers [2]. The risk factors for ovarian cancer include family history of ovarian carcinoma and genetic mutations. Ovarian cancer has a disproportionately poor survival rate due to the late onset of symptoms. Increased understanding of the relevant molecular mechanisms that regulate ovarian carcinogenesis may result in improved methods of diagnosis, treatment, and prevention.

*Ras* homolog gene family member A (*RhoA*) is a small (~22 kDa) G protein/guanosine triphosphatase that is part of the Ras-related C3 botulinum toxin substrate (Rac) subfamily of the Rho family [3]. *RhoA* can reorganize the cell cytoskeleton and regulate cell migration by activating effector proteins such as Rho-associated coiled-coil kinase (ROCK) [4]; such changes are associated with tumor invasion and migration in several types of carcinoma cells [5,6]. Phosphoinositide 3-kinase/protein kinase B (PI3K/Akt)-dependent phosphorylation of glycogen synthase kinase-3β (GSK3β) and RhoA activation regulate Wnt-5a–induced gastric cancer cell migration [7], in line with results for breast cancer cells [8]. *Wnt-5a* is a representative ligand for the seven-transmembrane receptor frizzled-5 and the tyrosine kinase orphan receptor 2, activating β-catenin–independent pathways and regulating a variety of cellular functions, such as proliferation, differentiation, migration, adhesion, and polarity [9]. Marked expression of Wnt-5a has previously been reported in human metastatic melanoma and a variety of human primary tumor samples. However, the exact nature of its role in ovarian carcinoma remains undetermined.

To clarify the role of *RhoA* and *Wnt-5a* in ovarian carcinogenesis and subsequent progression, we examined their mRNA and protein expression in normal fallopian tube epithelium, benign tumors, primary ovarian carcinomas, and metastatic ovarian carcinomas simultaneously, the first time such an investigation has been carried out, and compared them with the clinicopathological features of patients with ovarian carcinoma. To study the molecular mechanisms, we observed the effects of RhoA or Wnt-5a knockdown on the phenotypes and expression of molecules regulated in ovarian carcinoma cells *in vitro*.

## 2. Results

### 2.1. Correlation of RhoA and Wnt-5a mRNA and Protein Expression with Pathogenesis and Aggressiveness of Ovarian Carcinoma

**Figure 1.**
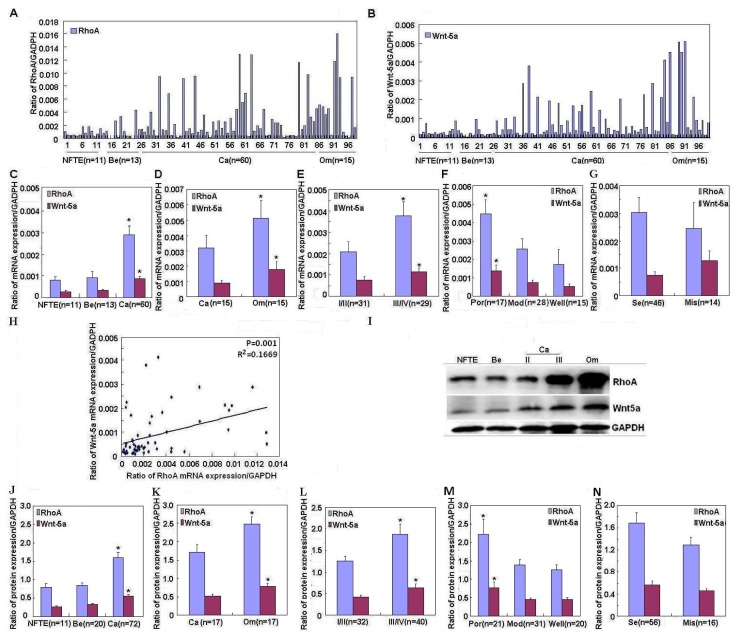
Correlation of RhoA and Wnt-5a mRNA and protein expression with the pathogenesis and aggressiveness of ovarian carcinoma. (**A**–**G**,**I**–**M**) RhoA and Wnt-5a mRNA and protein expression quantified in normal fallopian tube epithelium (NFTE), benign tumors (Be), primary ovarian carcinomas (Ca), and metastatic omentum (Om) using real-time PCR (**A**,**B**) and western blotting (**I**); Significantly higher RhoA and Wnt-5a mRNA and protein expression found in ovarian carcinomas compared to benign tumors or normal fallopian tube epithelium (**C**,**J**) and in metastatic omentum foci than in ovarian carcinomas (**D**,**K**); Positive association between RhoA and Wnt-5a mRNA and protein expression levels with FIGO staging (**E**,**L**, stage I/II *vs*. stage III/IV) and degree of differentiation (**F**,**M**, poorly differentiated (Por) *vs*. moderately differentiated (Mod) and well differentiated (Well)) in ovarian carcinoma; (**G**,**N**) No correlations between RhoA or Wnt-5a mRNA and protein expression and pathological subtype in ovarian carcinoma; and (**H**) Significant positive correlation between *RhoA* mRNA expression and *Wnt-5a* mRNA expression in ovarian carcinoma (*R*^2^ = 0.1669). *p < 0.05.

RhoA and Wnt-5a mRNA and protein expression were quantified in normal fallopian tube epithelium, benign cystadenoma (serous and mucinous), primary ovarian carcinomas, and metastatic omentum using real-time polymerase chain reaction (PCR) (Figure 1A,B) and western blotting (Figure 1I). RhoA mRNA and protein expression were significantly higher in ovarian carcinomas than in benign tumors or normal fallopian tube epithelium (Figure 1C,J, *p* < 0.05) and in metastatic omentum foci than in ovarian carcinomas (Figure 1D,K, *p* < 0.05); similar trends were observed for Wnt-5a, more details can be found in Tables S1 and S2.

Both RhoA and Wnt-5a mRNA and protein expression were positively associated with International Federation of Gynecology and Obstetrics (FIGO) stage (III/IV *vs*. I/II; Figure 1E,L, *p* < 0.05) and degree of differentiation in ovarian carcinoma (poorly differentiated carcinomas *vs*. other subtypes; Figure 1F,M, *p* < 0.05). However, no correlations were observed between RhoA or Wnt-5a expression and pathological subtype in ovarian carcinoma (Figure 1G,N, *p* > 0.05). *RhoA* mRNA expression was significantly positively correlated with *Wnt-5a* mRNA expression in ovarian carcinoma (Figure 1H, *p* = 0.001, *R*^2^ = 0.1669).

### 2.2. Effects of RhoA or Wnt-5a Knockdown on Phenotype and Expression of Wnt-5a Signaling-Related Molecules in Ovarian Carcinoma Cells

**Figure 2.**
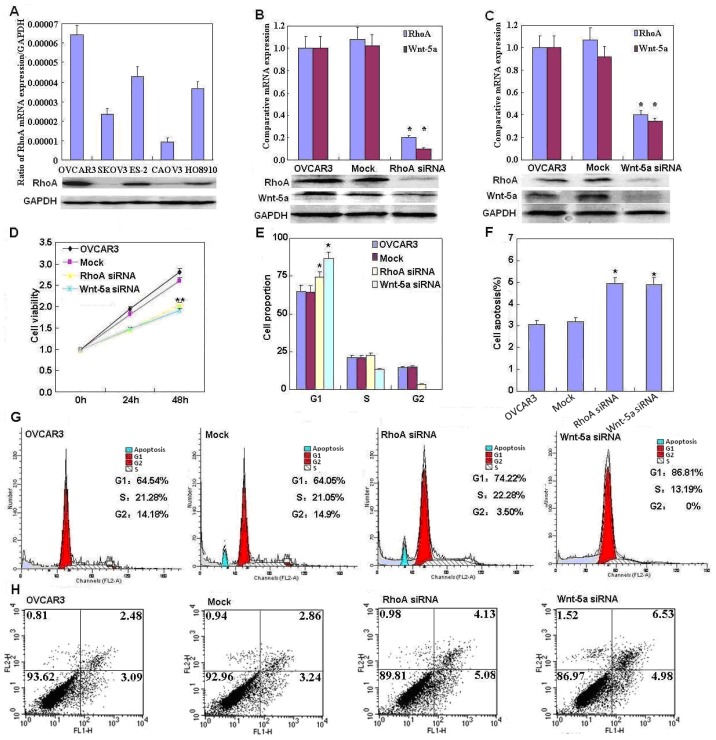
Effects of RhoA or Wnt-5a knockdown on mRNA and protein expression, cell proliferation, cell cycle, and apoptosis of ovarian carcinoma cells. (**A**) OVCAR3 cells were used for RhoA siRNA transfection because RhoA mRNA and protein expression was highest in this cell line; RhoA siRNA (**B**) or Wnt-5a siRNA transfection (**C**) significantly reduced RhoA and Wnt-5a expression in OVCAR3 cells compared to negative control (NC) or mock-transfected cells. OVCAR3 cells transfected with RhoA siRNA or Wnt-5a siRNA exhibited significantly reduced cell viability (**D**) in comparison with NC and mock-transfected cells; (**E**,**G**) Significantly increased G_1_ arrest in OVCAR3 cells transfected with RhoA siRNA or Wnt-5a siRNA in comparison with NC and mock-transfected cells; and (**F**,**H**) Significantly increased apoptosis in OVCAR3 cells transfected with RhoA siRNA or Wnt-5a siRNA in comparison with NC and mock-transfected cells. *p < 0.05. Results are representative of three separate experiments; data are expressed as the mean ± standard deviation.

**Figure 3.**
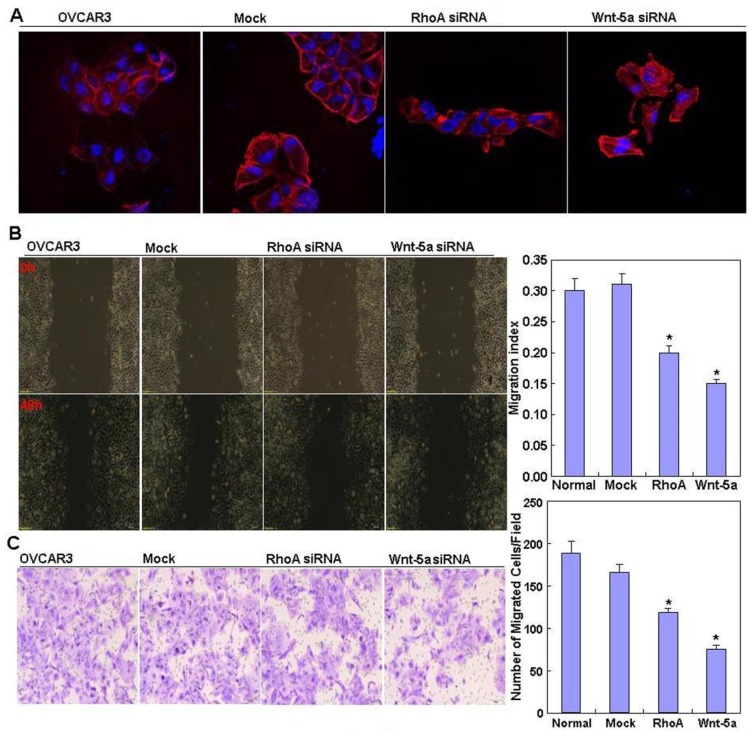
Effects of RhoA or Wnt-5a knockdown on the invasive ability of OVCAR3 cells. (**A**–**C**) OVCAR3 cells transfected with RhoA siRNA or Wnt-5a siRNA exhibited suppressed lamellipodia formation (**A**) magnification ×40; significantly reduced migration (**B**), Scale bar = 200 μm; and significantly reduced cell invasion (**C**) in comparison with NC and mock-transfected cells, magnification ×20. *p < 0.05. Results are representative of three separate experiments; data are expressed as the mean ± standard deviation.

**Figure 4.**
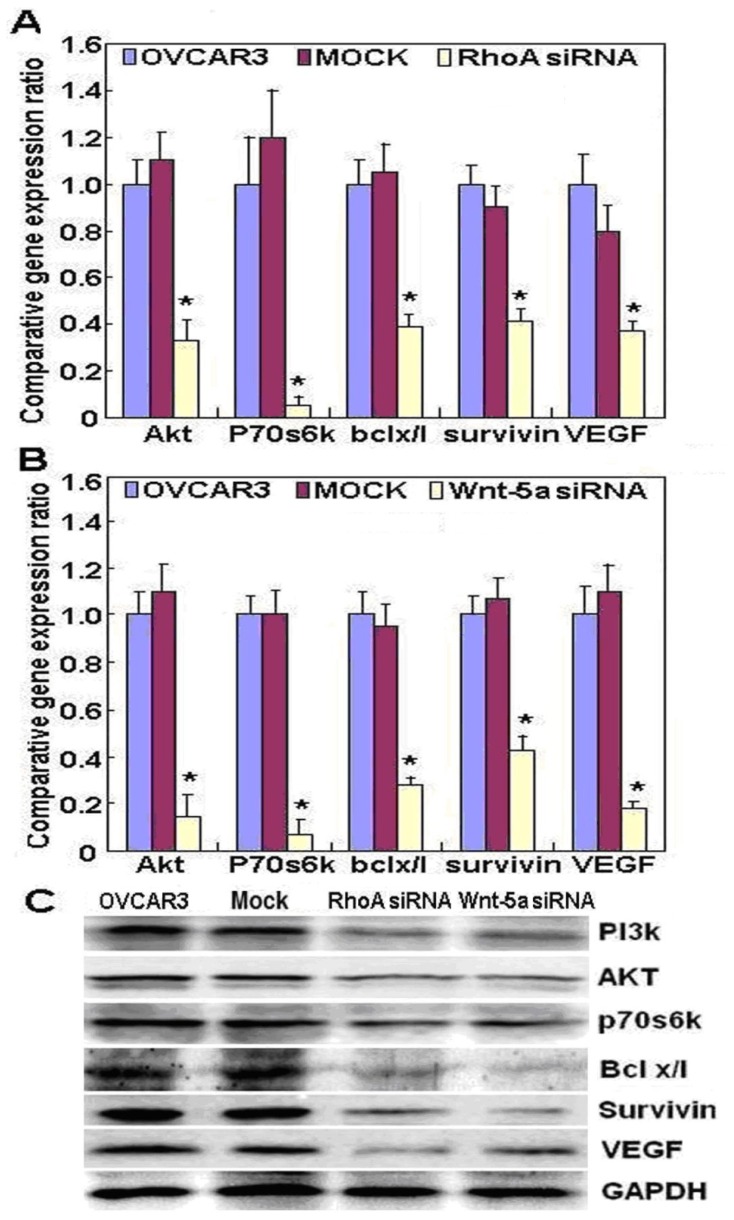
OVCAR3 cell mRNA and protein expression profiles after RhoA or Wnt-5a knockdown. (**A**–**C**) OVCAR3 cells transfected with RhoA siRNA or Wnt-5a siRNA expressed significantly lower levels of Akt, Bcl-xL, VEGF, p70S6k, and survivin mRNA (**A** and **B**) and protein (**C**) in comparison with NC and mock-transfected cells. *p < 0.05.

Figure 2A shows that RhoA mRNA and protein expression was highest in OVCAR3 cells; therefore, we used OVCAR3 cells for RhoA small interfering RNA (siRNA) transfection. RhoA and Wnt-5a mRNA and protein expression in OVCAR3 RhoA or Wnt-5a siRNA transfectants were significantly reduced as compared to negative control (NC) and mock-transfected cells (Figure 2B,C, *p* < 0.05), as demonstrated by real-time PCR and western blotting. Both RhoA and Wnt-5a siRNA transfectants exhibited significantly slower growth than NC and mock-transfected cells (Figure 2D, *p* < 0.05), based on the CCK-8 assay. Propidium iodide (PI) staining and flow cytometry revealed significant induction of G_1_ arrest in RhoA and Wnt-5a siRNA transfectants (Figure 2E,G, *p* < 0.05). Knockdown of either RhoA or Wnt-5a induced significantly higher levels of apoptosis (Figure 2F,H, *p* < 0.05), indicated by annexin V–fluorescein isothiocyanate (FITC) staining; suppressed lamellipodia formation (Figure 3A), indicated by loss of F-actin structure in cells stained with Alexa Fluor^®^ (Invitrogen, Carlsbad, CA, USA) 568 phalloidin; and reduced cell migration in the wound healing assay (Figure 3B, *p* < 0.05) and invasion in the Transwell invasion assay (Figure 3C, *p* < 0.05) in comparison with NC and mock-transfected cells. In cells transfected with RhoA siRNA, there was reduced expression of Akt, Bcl-xL, vascular endothelial growth factor (VEGF), p70 S6 kinase (p70S6k), and survivin mRNA in OVCAR3 cells (Figure 4A,B, *p* < 0.05); similar effects were observed in cells transfected with Wnt-5a siRNA. Western blotting showed that knockdown of either RhoA or Wnt-5a also downregulated the levels of Akt, PI3K, Bcl-xL, VEGF, p70S6k and survivin protein expression (Figure 4C).

## 3. Discussion

As reviewed by Hall [4], *RhoA* exerts multiple functions in tumor metastasis by orchestrating the action of multiple downstream effectors and promoting the degradation and reconstruction of the extracellular matrix. Ellenbroek *et al*. [10] reported that RhoA protein was overexpressed in head and neck cancers and gastric cancer, indicating that RhoA upregulation is closely associated with carcinogenesis. Our study showed that *RhoA* expression was significantly upregulated in ovarian carcinomas compared to normal fallopian tube epithelium and benign tumors, in accordance with Horiuchi *et al*. [11]. However, *RhoA* expression was upregulated in metastatic omentum compared to ovarian carcinomas; additionally, *RhoA* expression was positively associated with FIGO stage and degree of differentiation in ovarian carcinoma. These findings indicate that *RhoA* overexpression might affect ovarian carcinogenesis and subsequent progression.

In addition, we found that RhoA knockdown induced G_1_ arrest and apoptosis, decreased lamellipodia formation, and reduced cell migration and invasion, which indicates that RhoA downregulation may suppress the aggressive phenotypes of ovarian carcinoma cells. Our data are in agreement with that of Peacock *et al*. [12], who reported that *RhoA* promotes reorganization of the actin cytoskeleton, regulates cell shape and attachment, and coordinates cell motility and actomyosin contractility. We also found that knockdown of RhoA significantly reduced both mRNA and protein expression of Akt, p70S6k, Bcl-xL, survivin, and VEGF in OVCAR3 cells. According to the literature, these molecules are involved in the regulation of proliferation, apoptosis, invasion, and metastasis of cancer cells [13–15]. Taken together, these results suggest that *RhoA* may promote proliferation, anti-apoptosis, and invasion by modulating the expression of these genes.

*Wnt-5a* is a prototypic ligand that activates a β-catenin–independent pathway in Wnt signaling. *Wnt-5a* has been demonstrated to exert differential effects on cancer development. It promotes cancer progression and metastasis in malignant melanoma, breast cancer, and gastric cancer [8,16–18]. Kurayoshi *et al*. [18] reported that *Wnt-5a* stimulated cell motility, invasiveness, and aggressiveness in some cancer cells, suggesting that it exerts oncogenic effects. Li *et al*. [19] also reported that *Wnt-5a* exerted stem cell functions in lung cancer. In contrast, some reports indicate that in view of its ability to inhibit cell proliferation, motility, or invasion in thyroid tumor and colorectal cancer cell lines, *Wnt-5a* acts as a tumor suppressor [20–24]. Our study showed that *Wnt-5a* was also significantly upregulated in ovarian carcinomas compared to normal fallopian tube epithelium and benign tumors and in metastatic omentum compared to primary carcinomas, in line with Peng *et al*. [25] and Badiglian *et al*. [26], indicating that *Wnt-5a* may be involved in the tumorigenesis and progression of ovarian cancer. The functional experiments also suggested that Wnt-5a knockdown attenuated the aggressiveness of ovarian carcinoma, including proliferation, anti-apoptosis, migration, and invasion, by modulating phenotype-related molecules such as *Akt*, *PI3K*, *Bcl-xL*, *VEGF*, *p70S6k*, and *survivin*.

Although Strutt *et al*. [27] reported that Wnt-mediated cell migration appeared to require RhoA activation, and Qian *et al*. [28] found that *Wnt-5a* activated planar cell polarity (PCP) through a RhoA-dependent process, leading to control of cellular movement, we found that Wnt-5a knockdown significantly reduced both RhoA mRNA and protein expression. *RhoA* mRNA expression was positively correlated with that of Wnt-5a mRNA in ovarian carcinoma tissue. These results suggest that there may be a positive correlation between *RhoA* and *Wnt-5a* expression in ovarian carcinoma. However, further study of validation in *in vitro* models may better explain the obtained results.

In conclusion, we demonstrated for the first time that *RhoA* and *Wnt-5a* may be associated with ovarian carcinogenesis and apoptosis inhibition; there might be positive correlation between RhoA and Wnt-5a expression. *RhoA* has potential as a biomarker of tumorigenesis, differentiation, and progression in ovarian carcinoma.

## 4. Patients and Methods

### 4.1. Cell Culture and Transfection

The ovarian carcinoma cell lines CAOV3 (serous adenocarcinoma), OVCAR3 (serous cystic adenocarcinoma), SKOV3 (serous papillary cystic adenocarcinoma), HO8910 (serous cystic adenocarcinoma), and ES-2 (clear cell carcinoma) were purchased from ATCC (Manassas, VA, USA). They were maintained in RPMI 1640 (ES-2, HO8910, OVCAR3), DMEM (CAOV3), or McCoy’s 5A (SKOV3) media supplemented with 10% fetal bovine serum (FBS), 100 units/mL penicillin, and 100 μg/mL streptomycin in a humidified atmosphere of 5% CO_2_ at 37 °C. OVCAR3 cells were transfected with RhoA siRNA or Wnt-5a siRNA (Sigma-Aldrich, St. Louis, MO, USA). The *RhoA* target sequences were 5′-GUUUAUUAAUCUUAGUGUAdTdT-3′ (sense) and 5′-UACACUAAGAUUAAUAAACdTdT-3′ (antisense). The *Wnt-5a* target sequences were 5′-CUGACUACUGCGUGCGCAAdTdT-3′ (sense) and 5′-UUGCGCACGCAGUAGUCAGdTdT-3′ (antisense). The NC siRNA sequences were 5′-UUCUCCGAACGUGUCACGUTT-3′ (sense) and 5′-ACGUGACACGUUCGGAGAATT-3′ (antisense). Transfected cells were collected by centrifugation, rinsed with phosphate-buffered saline (PBS), and total proteins were extracted by sonication in radioimmunoprecipitation assay (RIPA) buffer.

### 4.2. Cell Viability Assay

Cell Counting Kit-8 (CCK-8; Dojindo, Tokyo, Japan) was used to determine the numbers of viable cells. Briefly, 2.0 × 10^3^ cells/well were seeded into 96-well plates and allowed to adhere. The next day, the media was changed to starvation medium (total volume 100 μL/well) for an additional 24 h, and cells were challenged with 200 ng/mL RhoA/Wnt-5a/Mock siRNAs (R & D Systems, Minneapolis, MN, USA). At the appropriate time points, 10 μL CCK-8 solution was added to each well, the plates incubated for 4 h, and the absorbance values measured at 450 nm using a microplate reader.

### 4.3. Cell Cycle Analysis

After incubation for 48 h at 37 °C in an atmosphere of 5% CO_2_, cells were trypsinized, harvested, washed twice in PBS, and fixed in 10 mL ice-cold ethanol for at least 2 h. Cells were then washed twice in PBS and incubated with 500 μL RNase (0.25 mg/mL) at 37 °C for 30 min. Cells were pelleted by centrifugation, resuspended in 50 μg/mL Propidium Iodide (PI, KeyGen, Nanjing, China), and incubated at 4 °C in the dark for 30 min. The PI signal was examined using flow cytometry.

### 4.4. Flow Cytometric Apoptosis Assay

Flow cytometry was performed using cells stained with PI and FITC-labeled annexin V (KeyGen, Nanjing, China) to detect phosphatidylserine externalization as an endpoint indicator of early apoptosis, as described in the manufacturer’s instructions. Briefly, after incubation for 48 h at 37 °C in an atmosphere of 5% CO_2_, cells were washed twice with cold PBS, resuspended in 1× binding buffer at 1 × 10^6^ cells/mL, and incubated with 200 μL Binding Buffer and 10 μL Annexin V–FITC. Samples were gently vortexed, incubated for 15 min at 25 °C in the dark, 300 μL Binding Buffer and 5 μL PI were added to each tube, and flow cytometry was performed within 1 h.

### 4.5. Wound Healing Assay

Cells were seeded at 1.0 × 10^6^ cells/well in 6-well culture plates. After cells had grown to confluence, the confluent monolayer in each well was scratched with a pipette tip, washed three times with PBS, and cultured in FBS-free medium. Cells were photographed at 0, 12, 24, 48 h (*n* = 9); the scratch area was measured using Image J software (National Institutes of Health, Bethesda, MD, USA). The wound healing rate = (Area of original wound − Area of actual wound at different times)/Area of original wound × 100%.

### 4.6. Cell Invasion Assay

A thin layer of Matrigel (40 μL from 8 mg/mL stock solution; Becton-Dickinson Labware, Bedford, MA, USA) was overlaid on the upper surface of 6.5-mm Transwell chambers (8-μm pore size; BD Bioscience, San Jose, CA, USA). The Matrigel was allowed to solidify by incubating the plates for 4 h at 37 °C. Culture medium was added to the bottom Transwell chamber. Cells were resuspended in serum-free RPMI 1640 (HyClone, Logan, UT, USA) at 2.5 × 10^5^ cells/mL, and 5 × 10^4^ cells were added to the top Transwell chambers. Following 48-h incubation, cells that had not invaded through the Matrigel were removed from the upper surface using cotton swabs. Cells that had invaded through the Matrigel and reached the bottom surface of the filters were fixed in methanol and stained with 0.1% crystal violet. Invasion was quantified by counting the number of cells under a Olympus fluorescence microscope (Tokyo, Japan) equipped with a 16-square reticle. The surface area of this grid was 1 mm^2^. Five separate fields were counted for each filter (*n* = 3); the total numbers of cells were compared among experimental groups using the *t*-test with the assumption of two-tail distribution and two samples with equal variance. A difference of *p* < 0.05 was considered statistically significant.

### 4.7. Immunofluorescent Staining

Cells were grown on glass coverslips, fixed with PBS containing 4% formaldehyde for 10 min, permeabilized with 0.2% Triton X-100 in PBS for 10 min at room temperature, washed with PBS, and incubated with Alexa Fluor^®^ 568 phalloidin (Invitrogen, Carlsbad, CA, USA) overnight at 4 °C to visualize the lamellipodia. Nuclei were stained with 1 μg/mL diamidino phenylindole (Sigma-Aldrich, St. Louis, MO, USA) for 15 min at 37 °C. Coverslips were mounted using SlowFade^®^ Gold antifade reagent (Invitrogen, Carlsbad, CA, USA) and cells were observed using a laser confocal microscope (Olympus, Tokyo, Japan).

### 4.8. Ovarian Epithelium and Carcinoma Samples

Between August 2003 and December 2011, normal fallopian tube epithelium, ovarian epithelial benign tumors (serous and mucinous cystadenoma), primary carcinoma specimens, and metastatic omentum were obtained from patients undergoing surgical resection at the Department of Gynecology, The First Affiliated Hospital of China Medical University. The tumor specimens were microscopically confirmed by pathologists. The average age at surgery was 51.6 years (range 20–81 years). Each ovarian carcinoma specimen was evaluated according to the International Federation of Gynecology and Obstetrics (FIGO) staging system (2009). The histological architecture of ovarian carcinoma was defined in terms of World Health Organization classification. Samples were frozen immediately in liquid nitrogen and stored at −80 °C until analysis. None of the patients underwent chemotherapy, radiotherapy, or adjuvant treatment before surgery. Informed consent was obtained from all subjects; the China Medical University Ethics Committee approved the study.

### 4.9. Real-Time Reverse Transcriptase PCR

Total RNA was extracted from ovarian carcinoma cell lines and ovarian tissues using TRIzol (Takara, Shiga, Japan) according to the manufacturer’s protocol. Total RNA (2 μg) was reverse transcribed to complementary DNA (cDNA) using avian myeloblastosis virus transcriptase and random primers (Takara, Shiga, Japan). The oligonucleotide primers for PCR were based on GenBank sequences (Table S3). Real-time PCR amplification of the cDNA was performed in 20-μL reactions according to the SYBR Premix Ex Taq™ II kit (Takara, Shiga, Japan); glyceraldehyde-3-phosphate dehydrogenase (*GAPDH*) was used as the internal control.

### 4.10. Western Blotting

Protein was extracted in ice-cold RIPA lysis buffer and its concentration was determined by protein assay kit (Bio-Rad Laboratories, Hercules, CA, USA). Denatured proteins (100 μg) were separated on sodium dodecyl sulfate–polyacrylamide gels, transferred to Hybond membranes (Amersham, Munich, Germany), and blocked overnight in 5% skimmed milk in Tris-buffered saline with Tween 20 (TBST). For immunoblotting, membranes were incubated for 15 min with antibodies against RhoA (Abcam, Cambridge, UK), Wnt-5a, PI3K, Akt, Bcl-xL, survivin, VEGF (Santa Cruz Biotechnology, Santa Cruz, CA, USA), or p70s6k (T421/s424; Cell Signaling Technology, Danvers, MA, USA). Then, the membranes were rinsed with TBST and incubated with anti-mouse, anti-rabbit, or anti-goat IgG antibodies conjugated to horseradish peroxidase (1:1000; Dako, Carpinteria, CA, USA) for 15 min. All incubations were performed in a microwave oven (Oriental Rotor, Tokyo, Japan) with intermittent irradiation. Bands were visualized on X-ray film (Fujifilm, Tokyo, Japan) using ImageQuant LAS 4000 (Fujifilm, Tokyo, Japan) and ECL Plus detection reagents (Santa Cruz Biotechnology, Santa Cruz, CA, USA). Subsequently, the membranes were washed with WB Stripping Solution (pH 2–3; Nacalai Tesque, Tokyo, Japan) for 1 h and reprobed using mouse anti-GAPDH antibody (Sigma-Aldrich, St. Louis, MO, USA) as the internal control.

### 4.11. Statistical Analysis

Statistical analysis was performed using the Spearman correlation test for ranked data and the Mann–Whitney *U* test and paired samples *t*-test to compare the means of different groups. *p <* 0.05 were considered statistically significant. SPSS 10.0 software (SPSS, Chicago, IL, USA) was used to analyze all data.

## 5. Conclusions

Our findings indicate that RhoA and Wnt-5a are associated with ovarian carcinogenesis and apoptosis inhibition; there might be a positive correlation between RhoA and Wnt-5a expression. RhoA is a potential tumorigenesis, differentiation, and progression biomarker in ovarian carcinoma.

## Supplementary Information
