# Supplementary material for: The Involvement of RhoA and Wnt-5a in the Tumorigenesis and Progression of Ovarian Epithelial Carcinoma
Source: Int J Mol Sci. 2013 Dec 12;14(12):24187–99. doi: 10.3390/ijms141224187 (PMC3876104; doi:10.3390/ijms141224187)
Supplement: Supplementary file 1 [file ijms-14-24187-s001.pdf]

Supplementary Information

Table S1. Correlation of *RhoA* and *Wnt-5a* mRNA expression with tumorigenesis and aggressiveness of ovarian carcinoma.

Clinicopathological features	*n*	***RhoA* mRNA expression/GAPDH**	***Wnt-5a* mRNA expression/GAPDH**
**Pathological classification**			
Serous adenocarcinoma	46	0.003032 ± 0.003243	0.000747 ± 0.000736
Miscellaneous subtypes	14	0.002432 ± 0.003582	0.001261 ± 0.001421
**FIGO staging**			
I–II	31	0.002067 ± 0.002787	0.000749 ± 0.000856
III–IV	29	0.003773 ± 0.003621 *	0.001144 ± 0.000985 *
**Differentiation**			
Well	15	0.001717 ± 0.003162	0.000516 ± 0.000575
Moderate	28	0.002559 ± 0.002976	0.000733 ± 0.000874
Poor	17	0.004476 ± 0.003522 **	0.001396 ± 0.001147 **

* Compared with International Federation of Gynecology and Obstetrics (FIGO) stage I/II (p = 0.0445; p = 0.0279); ** Compared with moderately differentiated and well differentiated (p = 0.0182; p = 0.0057).

Table S2. Correlation of RhoA and Wnt-5a protein expression with tumorigenesis and aggressiveness of ovarian carcinoma.

Clinicopathological features	*n*	***RhoA* protein expression/GAPDH**	***Wnt-5a* protein expression/GAPGH**
**Pathological classification**			
Serous adenocarcinoma	56	1.684880 ± 1.400929	0.563563 ± 0.489970
Miscellaneous subtypes	16	1.285997 ± 0.589267	0.452062 ± 0.181456
**FIGO staging**			
I–II	32	1.251530 ± 0.698392	0.421421 ± 0.252762
III–IV	40	1.872007 ± 1.545606 *	0.632676 ± 0.532978 *
**Differentiation**			
Well	20	1.259664 ± 0.617077	0.452474 ± 0.202293
Moderate	31	1.396119 ± 0.785278	0.441835 ± 0.268450
Poor	21	2.212203 ± 1.973854 **	0.764103 ± 0.685803 **

* Compared with International Federation of Gynecology and Obstetrics (FIGO) stage I/II (p = 0.0390; p = 0.0429); ** Compared with moderately differentiated and well differentiated (p = 0.007514; p = 0.004691).

Table S3. RT-PCR primers.

Gene	**Primer sequence**	**Target sequence**	**AT (°C)**	**Product size (bp)**	**Extension time (s)**
RhoA	F: 5'-ACTCGGATTCGTTGCC-3'<br>R: 5'-TTGGGACAGAAATGCTTGA-3'	NM-001664<br>585-860	60	276	34
Wnt-5a	F: 5'-GCGAAGACAGGCATCAAA-3'<br>R: 5'-CCTTGGCAAAGCGGTAG-3'	NM-003392<br>946-1240	60	295	34
P70S6k	F: 5'-TAAAGCATCCCTTCATCG-3'<br>R: 5'-CAGGCAGTGTCTTCCATAA-3'	NM_003161<br>552-690	60	139	34
PI3K	F: 5'-CACCGCATTTGTCGT-3'<br>R: 5'-CTCCCACTTCTACGC-3'	NM_001242466<br>4016-4240	60	225	34
Akt	F: 5'-GGGACAGAGGAGCAAGGT-3'<br>R: 5'-CGACAGCGGAAAGGTTAA-3'	NM_005163<br>2656-2863	60	208	34
Bcl/xL	F: 5'-TTCCCAGAAAGGATACAGC-3'<br>R: 5'-GGGTCTCCATCTCCGATT-3'	Z23115<br>185-276	60	92	34
Survivin	F: 5'-CTTGGCCCAGTGTTTCTT-3'<br>R: 5'-GCTTCCAGTCCCTCCCT-3'	DQ227257<br>159-282	60	124	34
VEGF	F: 5'-AGGAGGAGGGCAGAATC-3'<br>R: 5'-ATGTGCTGGCCTTGGT-3'	NM_001171630<br>1131-1388	60	258	34
GAPDH	F: 5'-CAATGACCCCTTCATTGACC-3'<br>R: 5'-TGGAAGATGGTGATGGGATT-3'	NM_002046.3<br>201-335	60	135	34

AT = annealing temperature.

© 2013 by the authors; licensee MDPI, Basel, Switzerland. This article is an open access article distributed under the terms and conditions of the Creative Commons Attribution license (http://creativecommons.org/licenses/by/3.0/).
